# Foreign body in the thoracic spine due to a nail gun penetrating injury: A case report

**DOI:** 10.1097/MD.0000000000013870

**Published:** 2018-12-28

**Authors:** Chi-Wei Chen, Shih-Chia Yang, Kuan-Ting Liu, Yen-Hung Wu

**Affiliations:** a Department of Emergency Medicine, Kaohsiung Medical University Hospital; b School of Medicine, College of Medicine, Kaohsiung Medical University, Kaohsiung, Taiwan.

**Keywords:** foreign body, nail gun injury, thoracic spine

## Abstract

**Rationale:**

Spinal cord injuries could be catastrophic because they may result in severe neurovascular complications. Here, we present a case of thoracic spine-penetrating injury by a nail-gun.

**Patient concerns:**

A 60-year-old male presented to our emergency department with complaints of progressive right chest pain for 1 week that was preceded by back pain. He had a medical history of hypertension and denied any trauma history. He had alert consciousness and stable vital signs. He was a carpenter. Upon physical and neurological examination, no obvious wounds or vesicle formation were noted, and the patient was neurologically intact.

**Diagnosis:**

Laboratory test results showed abnormally elevated D-dimer levels. Electrocardiography showed normal sinus rhythm. Chest radiography showed no mediastinal widening. Chest computed tomography was performed. The formal radiology report indicated a foreign body in the T4-5 spinal cord and upper back.

**Interventions:**

A neurosurgeon was consulted with suggestion of operation. We performed T4-5 laminectomy and foreign body removal. The foreign body, stuck to the spinal cord with dural rupture, was removed and found to be a 5 cm-long broken nail.

**Outcomes:**

The pain resolved immediately post operation.

**Lessons:**

Surgical removal of the foreign body is recommended if neurovascular complications or cerebrospinal fluid (CSF) leak is detected. Obtaining the patient's complete history, including occupation, might be helpful in determining the diagnosis. Careful interpretation of diagnostic imaging is necessary for avoiding medical disputes. Even in the absence of wounds and ecchymosis, trauma-related injury should be considered.

## 1 Introduction

Spinal cord injuries could be catastrophic because they may result in severe neurovascular complications, such as myelopathy, radiculopathy, and central nervous system infection.^[1]^ In literature review, the existing reports of nail-gun injury which has confirmed trauma history.^[2–9]^ The physician may miss the diagnosis of trauma-related injury if the patient did not describe a clear history of trauma event. In this case report, we present a patient with back pain who was unaware of trauma history and diagnosed with a penetrated nail-gun injury to the soft tissue of the back, T4 vertebral lamina, and spinal cord. After we contacted the regulations of institutional review board of the Kaohsiung Medical University Hospital, there was no need for ethical approval for this case report article. Informed consent was obtained from the patient.

## 2 Case report

**Figure 1:**
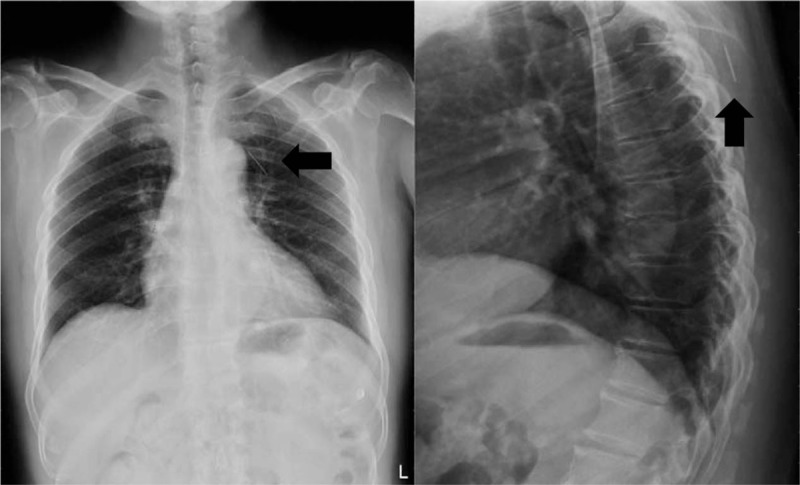
Chest X-ray scan showing foreign bodies (fragments of needle) in the spinal canal of T4-5 and upper back.

**Figure 2:**
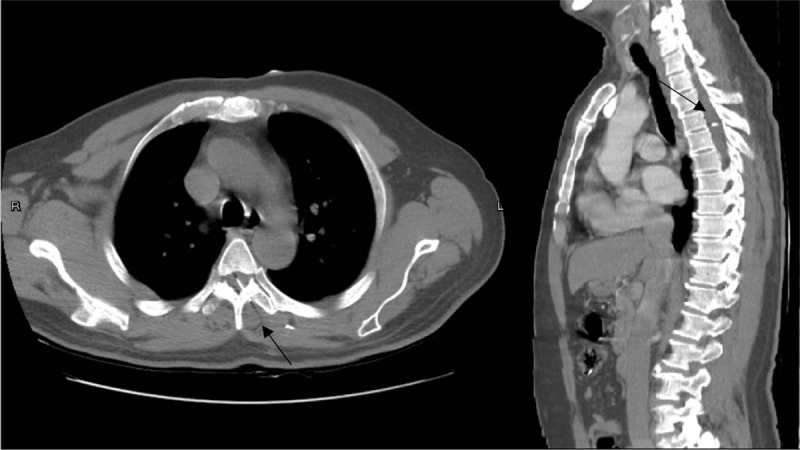
Computed tomography scan showing entrance of the nail through the left T5-6 spinal canal and soft tissue of the left back.

A 60-year-old male presented to our emergency department (ED) with complaints of progressive right chest pain for 1 week, that was preceded by back pain. He had a medical history of hypertension without regular control and denied any trauma history. He was alert, conscious with stable vital signs (body temperature, 37.6°C; pulse, 62 beats/min; respiratory rate, 18 breaths/min; and body pressure, 196/111 mmHg). He mentioned carpentry as his profession. Upon physical and neurological examination, no obvious wounds or vesicle formation were noted, and the patient was neurologically intact. Laboratory test results showed abnormally elevated D-dimer levels (2.01 mg/dL). Electrocardiography showed normal sinus rhythm. Chest radiography showed no mediastinal widening or obvious lesion. Chest computed tomography (CT) was performed to rule out acute cardiovascular and pulmonary diseases (e.g., aortic dissection and pulmonary embolism). The formal radiology report indicated a foreign body in the T4-5 spinal cord and upper back (Figs. 1 and 2). A neurosurgeon was consulted with suggestion of operation. We performed T4-5 laminectomy and foreign body removal. The foreign body, stuck to the spinal cord with dural rupture, was removed and found to be a 5 cm-long broken nail. The site was irrigated with antibiotic solution. Prophylactic antibiotics were administered for double protection against the possibility of developing meningitis. The pain resolved immediately post operation; no unusual events or neurological sequelae were observed at further follow-up.

## 3 Discussion

A patient's description of chest pain character may influence the emergency physician's differential diagnosis and the decision to rule out cardiopulmonary or gastrointestinal emergencies such as acute coronary syndrome, aortic dissection, pulmonary embolism, pneumothorax, and esophageal rupture. Otherwise, the differential diagnosis of non-traumatic shoulder or back pain includes musculoskeletal and neurological disorders. Referred pain from other sources, for example, acute myocardial disorders, cholecystitis, pancreatitis, and pelvic disorders, may also be considered. In our case, there was no obvious wound, and the patient also denied trauma history. Therefore, that misleads the emergency physician to consider internal medical problems only.

Spinal foreign bodies are related to either trauma or iatrogenic injuries. Bullets, knife blades, Kirschner wires, needles, glass or wooden fragments, or cotton gauzes have been reported as foreign bodies usually retained in the spinal canal.^[10]^ A nail gun is widely used at a work site, and frequently causes penetrating injuries owing to its high power and projectile speed (up to 100–150 m/s).^[11]^ A report estimated that an average of approximately 37,000 patients per year was treated for nail gun-related injuries in the ED from 2001 to 2005 in America.^[12]^ Only a few published reports regarding spinal nail gun-related injuries are available.^[2–9]^

A spinal foreign body may be fatal depending on its organic nature and the entry site; it may cause neurological complications or act as an infection source.^[13]^ Delayed neurological abnormalities have been reported, and may be triggered by a second minor injury. In our case, the exact time of injury was unknown. Therefore, careful interpretation of imaging is important.

X-ray imaging may miss detection of some radiolucent foreign bodies such as glass, wood, or cotton surgical gauzes. CT may help in detecting the foreign body and its trajectory. However, some foreign bodies may appear as hypodense lesions on the CT scan and may be confused for air. Some have recommended magnetic resonance imaging (MRI) for identifying the injury tract, cord or root lesions, and associated lesions including hematomas, disc herniation, and bone fragments.^[14]^ However, it may not be possible for all EDs to perform MRI urgently.

The treatment options included conservative approach and operation. Patients without neurovascular complications can be treated using the conservative approach; patients with neurovascular complications require the surgical approach. During operation, dural injury should be evaluated and high-volume of antibiotic irrigation should be administered.^[5]^ Patients must be monitored for cerebrospinal fluid (CSF) leak, infection, and vascular injury post-operatively.^[2]^

## 4 Conclusion

We present a relatively rare case of thoracic spine-penetrating injury by a nail-gun. Surgical removal of the foreign body is recommended if neurovascular complications or CSF leak is detected. Obtaining the patient's complete history, including occupation, might be helpful in determining the diagnosis. Careful interpretation of diagnostic imaging is necessary for avoiding medical disputes. Even in the absence of wounds and ecchymosis, trauma-related injury should be considered.

## Author contributions

**Resources:** Kuan-Ting Liu.

**Supervision:** Shih-Chia Yang, Kuan-Ting Liu.

**Visualization:** Shih-Chia Yang, Kuan-Ting Liu, Yen-Hung Wu.

**Writing – original draft:** Chi-Wei Chen.

**Writing – review & editing:** Yen-Hung Wu.
